# Self-medication of adults and children in Poland - results from outpatient health care physicians online questionnaire

**DOI:** 10.3389/fphar.2024.1413811

**Published:** 2024-08-13

**Authors:** Karolina Kłoda, Mateusz Babicki, Aleksander Biesiada, Małgorzata Gałązka-Sobotka, Iwona Kowalska-Bobko, Agnieszka Mastalerz-Migas

**Affiliations:** ^1^ MEDFIT Karolina Kłoda, Ul. Narutowicza, Szczecin, Poland; ^2^ Department of Family Medicine, Wroclaw Medical University, Wroclaw, Poland; ^3^ Polish Society of Family Medicine, Ul. Syrokomli, Wroclaw, Poland; ^4^ Institute of Healthcare Management, Lazarski University, Warsaw, Poland; ^5^ Faculty of Health Science, Institute of Public Health, Jagiellonian University Medical College, Cracow, Poland

**Keywords:** family medicine, GP, OTC, primary care, self-medication

## Abstract

**Introduction:** In Poland, the area of self-medication requires scientific and organizational evaluation. So far, no solutions sanctioning self-medication have been introduced. Therefore, the aim of this study was to recognize and analyze the practical experience of outpatient physicians regarding self-medication of their patients, as well as self-medication of children by their caregivers.

**Methods:** This study enrolled 386 participants and used a Computer-Assisted Web Interview that was disseminated online from 4th of July 2023 to 23rd of August 2023. The survey was addressed to outpatient healthcare physicians working in Poland.

**Results:** In doctors’ perspective the main three reasons for choosing self-medication in Poland were: taking advice from other people - family members or friends (59.1%), finding information regarding treatment online (52.9%) and ability to self-medicate in this kind of symptoms/disease (51.6%). Among adult patients, in 72.1% of cases, an independent decision to start antibiotic therapy was made. Such a decision occurred in 39.8% of pediatric patients. Children caregivers were more likely to visit the physician immediately with symptoms than in the case of adult patients (42.2% vs. 22.1%, *p* < 0.001).

**Conclusions:** Self-medication in Poland requires educational and organizational support at various levels - both social (information campaigns, school education), the healthcare system (increasing the role of medical professionals, including pharmacists), and finally in the area of legislation. Two areas seem to be particularly alarming - the use of antibiotics by adults and children and the mental health of both populations.

## Introduction

According to the WHO definition, self-medication includes the use of medical products by a patient to treat a disease or symptoms, or the self-administration of chronic medications prescribed by a doctor. It also includes self-recognition of the disease or symptoms, which the patient plans to treat. Responsible self-medication involves the use of over-the-counter (OTC) medications according to the label or the use of medications prescribed by a doctor. Irresponsible self-medication means incorrect use of the OTC medications, using expired or prescribed for other purposes medications, or sharing medications with others (WHO, 2000; Shehnaz et al., 2014; Ghodkhande et al., 2023).

Due to the healthcare systems overload during the COVID-19 pandemic, recommendations of social distancing and fear of infection, self-medication has become more common in many countries. According to the research conducted during the pandemic, the frequency of self-medication worldwide was nearly 45% and factors conducive to this behavior were recognized. The incidence was gender-related and increased with age. The most commonly purchased OTC medications were painkillers, vitamins and dietary supplements, anti-allergy drugs and cold and cough medicines (Shrestha et al., 2022). In the case of Poland, the frequency of *de novo* self-medication has increased (Makowska et al., 2020). The readiness to act on your own, was also high, long before the pandemic. A study from 2009 including data from 50 countries, found that 95% of respondents were open to the possibility of self-medication in minor diseases (The Nielsen Company, 2009; Stosic et al., 2011). Research conducted by the Substance Abuse and Mental Health Services Administration (SAMHSA) in the United States has shown that approximately three million people aged 12 and older have misused OTC medications at least once in their lives (Gonzales et al., 2011; Marathe et al., 2020).

Self-medication carries high risks, such as non intentional misuse or abuse of medications resulting from lack of knowledge. Moreover, the use of some drugs before medical consultation may mask the symptoms allowing obtaining the diagnosis. Especially that patients tend to not inform the physician about the medications they have taken, presenting sometimes new symptoms that can be linked with inappropriate self-medication. Another threat is that patients who take medications long-term because of chronic diseases are at risk of interactions with substances used in self-medication. And finally, drugs that have already been prescribed to a patient may not be properly stored until they are used again, which may result in a decrease or lack of their effect (Ruiz, 2010). These risks drive new research questions and scientific solutions. Recent studies focus on responsible self-medication with medical professional counseling in order to improve clinical effects and unburden healthcare systems. The role of the advising person can be performed by a physician, but by other healthcare workers (Rutter, 2015). The involvement of pharmacists could allow them to conduct consults and solve minor health problems of the patient in the pharmacy. Such an approach would be possible through following an appropriate algorithm, and then proposing treatment using OTC medications or explaining the administration of prescription drugs. However, such a solution needs educational and financial reinforcement (Alexa and Bertsche, 2023).

In Poland, the area of ​​self-medication requires scientific and organizational evaluation. Until now there has been no reliable data on the prevalence of self-medication. So far, no solutions sanctioning self-medication have been introduced, especially supported by the advice of a medical professional. Thus, there are no specific sites, like community pharmacies where an individual can receive an appropriate self-medication consult. We hypothesized that outpatient care (in particular primary healthcare - PHC) physicians are asked to support self-medication or to treat its negative effects and are able to indicate the patterns of their patients’ behavior. Family doctors and other PHC physicians in Poland are the first line medical proffesionnals accessible even on the same day of submitting a request for a consultation. Therefore, the aim of this study was to recognize and analyze the practical experience of outpatient physicians regarding self-medication of their patients, as well as self-medication of children by their caregivers.

## Materials and methods

The study was conducted by the Scientific Section of the Polish Society of Family Medicine together with the Institute of Healthcare Management at the Lazarski University. The research tasks discussed in this paper are part of the Self-medication Report, which is currently being developed. This study used a Computer-Assisted Web Interview (CAWI) that was disseminated online from 4th of July 2023 to 23rd of August 2023. The survey was addressed to doctors working in Poland. The survey was distributed via the social networking site Facebook.com within closed groups to which only doctors have access after verification of their professional medical license number. In addition, the mailing database of the Polish Society of Family Medicine was used. Before completing the form, participants were given the information regarding research objectives and methodology and were required to provide informed consent to access the questionnaire. Respondents could discontinue their participation at any time, without explanation. No personal information such as email addresses were collected to ensure anonymity.

Inclusion criteria for the study were: giving informed consent to participate in the study and being a outpatient physician. Failure to meet both criteria meant that no further participation in the study was possible.

A self-administered questionnaire was developed for the project, which consisted of closed-ended single- and multiple-answer questions. The first part of the questionnaire, which was a demographic interview, consisted of questions assessing socio-economic status, including age and gender. This was followed by questions assessing professional status, including career stage, length of service, main place of work (primary care, specialist clinic and hospital) and location of workplace. The main part of the survey consisted of interview regarding their personal experience with patients’ self-medication in both the children and the adults. The last part questioned for doctors’ opinions on self-medication and it is side effects management.

Assessment of physicians’ personal experience included indication from among the examples listed, in which situations their patients used self-medication. Possible answers included: headache, sore throat and pharyngitis, muscular-articular pain, cough, rhinitis, migraine, abdominal pain, back pain, digestive disorders (indigestion, diarrhea, constipation), menstrual pain, urinary tract infection, allergy, mental health disorders and other than those listed. Doctors were then asked to indicate how long, in their experience, patients use self-medication before they decide to consult a doctor, taking into account the time period: less than 3 days, 3–7 days, 7–14 days, 14 days to a month and more than a month. Then, for headache, respiratory tract infections (cough, rhinitis, pharyngitis), mental health problems, abdominal pain and urinary tract infections, doctors were asked to indicate the self-medication methods used by their patients from among the possible answers: home remedies, use of OTC medications, use of drugs previously prescribed by a doctor for similar complaints, staying at home and resting, waiting for symptoms to resolve on their own, implementing antibiotic therapy on their own, seeing a doctor immediately, other than those listed (multiple-answer question). Separate questions were developed for adult and pediatric patients.

In the final stage of the survey, respondents were asked to indicate potential reasons for self-medication of patients and to state whether they had encountered adverse effects following this behavior of their patients.

The study was conducted according to the guidelines of the Declaration of Helsinki and approved by the Bioethics Committee at Lazarski University (approval number: 04/06/23). The study, guided by the principle in scientific research to do no harm, gave the participants control over disclosure. Informed consent for research participation was obtained from all participants.

## Statistical analysis

The modified formula of Daniel and Cochran was used to calculate the minimum number of participants, for the total number of subjects, that is, 43130 primary care physicians, maintaining a 95% confidence interval (α = 0.95), a fraction size of 0.5 (unknown *a priori* response rate) (*p* = 0.5) and a maximum error of 5% (d = 0.05), the number of subjects could not be less than 381 (Windak et al., 2019).

The variables taken into the analysis had qualitative and quantitative character. The Shapiro-Wilk Test was used to assess the normality of the distribution. For qualitative variables, results were presented as %. For quantitative variables, basic descriptive statistics were presented. The Chi square test was used to assess statistical significance between quantitative variables. A *p*-value <0.05 was assumed to be statistically significant. The analysis was carried out using Statistica 13.0 by StatSoft.

## Results

### Characteristics of the study group

The study enrolled 386 physicians working in Poland, with an average age of 38.6 ± 9.5 years. Among the surveyed doctors, 87% work in PHC, 43.8% of the respondents hold the title of specialist in family medicine, and 30.2% are in the course of this specialization. The average seniority was 11.6 ± 9.3 years. A detailed description of the study group is presented in Table 1.

**TABLE 1:** Characteristics of the study group.

Variable		N (%)/M ± SD
Sex	Male	79 (20.5)
	Female	301 (78.0)
	Refusal to answer	6 (1.5)
Age	38.6 ± 9.5	
Medical specialization	Family medicine specialist	168 (43.8)
	Internal diseases specialist	44 (11.5)
	Pediatrician	26 (6.8)
	Other specialist	29 (7.6)
	Family medicine trainee	116 (30.2)
	Internal diseases trainee	5 (1.3)
	Pediatrics trainee	3 (0.8)
	Other specialization trainee	17 (4.4)
	Without specialization	34 (8.9)
Place of main work	City >500,000 residents	104 (27.1)
	City 50,000–500,000 residents	128 (33.3)
	City up to 50,000 residents	89 (23.2)
	Village	63 (16.4)
The main place of work	PHC	334 (87.0)
	Outpatient specialist care	9 (2.3)
	Other	41 (10.7)
Seniority		11.6 ± 9.3

M, mean; SD, standard deviation; N, number.

### Reasons for medical consultations after self-treatment

Among the analyzed answers, physicians most often indicate that their patients consult them because of the ineffectiveness of self-medication - 38.5%. For 33.3% of the cases, the reason was a greater severity of symptoms and 14.3% of patients did intend to self-medicate but did not know what treatment they should have started. Physicians observed that their patients most often base their knowledge of the treatment on previous medical recommendations - 62.5% and recommendations of family/friends - 59.6%. In 43.2% of cases, doctors declare that their patients gained knowledge from pharmacists. The list of answers is presented in Table 2.

**TABLE 2:** List of reasons for medical consultations and sources of knowledge about the recommended treatment among patients.

Question		N (%)
Why patients/parents/children caregivers choose self-medication and do not to consult a physician?	Other people: family, friends, advised them how to treat	227 (59.1)
	Found information about treatment on the Internet	203 (52.9)
	Usually in this kind of disease/symptoms they are able to cure the child/children or themselves	198 (51.6)
	They failed to make an appointment - the doctor was unavailable or the wait was too long	185 (48.2)
	The symptoms stopped before they thought they needed a consultation	145 (37.8)
	In this case, the advice of the pharmacist was enough for them	110 (28.6)
	Other reason	23 (6.0)
Reasons for medical consultation after attempting self-medication	Self-medication was ineffective	148 (38.5)
	Symptoms were more severe than usual	128 (33.3)
	Did not know how to self-medicate	55 (14.3)
	There was an open slot to see a physician	45 (11.7)
	Other people: family, friends, advised to go to the physician	4 (1.0)
	Other reason	4 (1.0)
Source of knowledge about the type of medication used	They were recommended by a physician once and since then I’ve been buying them myself	240 (62.5)
	They were recommended by a family/friend	229 (59.6)
	They always help in such a situation	188 (49.0)
	They found information about treatment on Internet	180 (46.9)
	They were recommended by a pharmacist	166 (43.2)
	The advertisement convinced them	123 (32.0)
	Other reason	8 (2.1)
In the last 2 years, have you experienced any side effects after self-medication among your patients?	Yes	264 (68.8)
	No	54 (14.1)
	I do not remember	66 (17.2)
Behavior of patients after adverse reactions (N = 264)	They consult a physician	250 (94.7)
	They stop taking the medicine	135 (51.1)
	They do nothing - wait for the side effect to stop on its own	32 (12.1)
	Contact the pharmacist	8 (3.0)
	Behave differently than indicated above	5 (1.9)
	Contact the medicine manufacturer	0 (0.0)
	They report to the Office for Registration of Medicinal Products, Medical Devices and Biocidal Products	0 (0.0)

In addition, 264 (68.8%) physicians report that they have encountered adverse reactions among their patients after self-medication implemented by them. Most often, these patients chose a medical consultation as a solution to their health problem - 94.7%. More than half (51.1%) decided to discontinue the drug, and in 12.1% of cases patients did nothing and waited for spontaneous resolution of symptoms. The results are summarized in Table 2.

### Self-medication among adult and pediatric patients

The analysis of questions regarding the reasons for self-medication by the patients showed that they were mostly significantly different in comparison of children and adults. In both groups, the most common cause of self-medication was cough (73.4% vs 88.5%, *p* < 0.001). Among adults, further causes included sore throat and pharyngitis, back pain, rhinitis and fever. In the case of children, apart from cough, their caregivers significantly more often attempted to self-medicate fever (82.8%), rhinitis (80.7%), abdominal pain (65.9%), and allergies (55.7%). On the other hand, adults more often attempt to self-medicate less specific symptoms, such as headache (54.4% vs. 19.0%, *p* < 0.001), muscle and joint pain (48.2% vs. 15.1%, *p* < 0.001) or low mood (15.4% vs. 5.1%, *p* < 0.001). A detailed list and comparison of the reasons for self-medication are presented in Table 3.

**TABLE 3:** Declared reasons for self-medication among patients of physicians in Poland, distinguishing between adult and pediatric patients.

Cause of self-medication	Adult N(%)	Child N(%)	*p*
Rhinitis	226 (58.9)	310 (80.7)	<0.001
Cough	282 (73.4)	340 (88.5)	<0.001
Fever	226 (58.9)	318 (82.8)	<0.001
Sore throat and pharyngitis	281 (73.2)	306 (79.7)	0.035
Headache	209 (54.4)	73 (19.0)	<0.001
Back pain	230 (59.9)	---	---
Abdominal pain	171 (44.5)	253 (65.9)	<0.001
Digestive disorders (indigestion, diarrhea, constipation, etc.)	201 (52.3)	152 (39.6)	<0.001
Muscle and joint pain	185 (48.2)	58 (15.1)	<0.001
Urinary system infection	190 (49.5)	115 (29.9)	<0.001
Allergy	140 (36.5)	214 (55.7)	<0.001
Migraine	69 (18.0)	---	---
Menstrual pain	63 (16.4)	25 (6.5)	<0.001
Low mood	59 (15.4)	21 (5.5)	<0.001
Other	39 (10.2)	42 (10.9)	0.732

N, number.

### Self-medication methods

When assessing the methods of self-medication, it was shown that in the case of headache, adults and children caregivers alike - most often reached for self-purchased OTC drugs. Moreover, adult patients were more likely to do nothing while waiting for the pain to subside on its own (46.1% vs. 18.5%, *p* < 0.001). In both cases home remedies were often used. Similarly, for sore throat, cough and rhinitis, self-purchased OTC medications were the most common treatment option for both groups of patients. Among adult patients, in 72.1% of cases, an independent decision to start antibiotic therapy was made. Such a decision occurred in 39.8% of pediatric patients. The surveyed physicians also observed that children caregivers were more likely to visit the physician immediately with symptoms than in the case of adult patients (42.2% vs. 22.1%, *p* < 0.001). Interestingly mental health disorders occurrence was left without any reaction by 73.2% adults vs. 64.8% of children caregivers (*p* < 0.0001). Nevertheless, in the case of children, 21.9% of physicians indicate that the caregivers went to the doctor immediately, while such a response was given only for 5.7% of adults. Table 4 presents a detailed list of self-medication methods with the distinction of headache, abdominal pain, symptoms of respiratory tract infection, urinary tract infection and mental health disorders.

**TABLE 4:** Self-medication methods for abdominal pain, symptoms of respiratory tract infections, urinary tract infections and mental health disorders, divided into adult and pediatric patients.

Cause of self-medication	Method	Adult N(%)	Child N(%)	*p*
Headache	Self-purchased OTC	353 (91.9)	283 (73.7)	<0.001
	Medications prescribed previously by a physician	171 (44.5)	90 (23.4)	
	Home methods	146 (38.0)	147 (38.3)	
	Rested, stayed at home	78 (20.3)	156 (40.6)	
	Immediately consulted the physician	42 (10.9)	147 (38.3)	
	Other	12 (3.1)	13 (3.4)	
	Didn’t do anything special, just waited for it to pass	177 (46.1)	71 (18.5)	
Rhinitis, cough, sore throat	Self-purchased OTC	345 (89.8)	292 (76.0)	<0.001
	Medications prescribed previously by a physician	165 (43.0)	224 (58.3)	
	Took an antibiotic available at home, recommended by a doctor for a previous infection/given by a friend	277 (72.1)	153 (39.8)	
	Home methods	298 (77.6)	247 (64.3)	
	Immediately consulted the physician	85 (22.1)	162 (42.2)	
	Rested, stayed at home	97 (25.3)	155 (40.4)	
	Didn’t do anything special, just waited for it to pass	116 (30.2)	30 (7.8)	
	Other	8 (2.1)	12 (3.1)	
Mental health	Self-purchased OTC	103 (26.8)	21 (5.5)	<0.001
	Medications prescribed previously by a physician	41 (10.7)	14 (3.6)	
	Home methods	26 (6.8)	26 (6.8)	
	Immediately consulted the physician	22 (5.7)	84 (21.9)	
	Rested, stayed at home	57 (14.8)	46 (12.0)	
	Didn’t do anything special, just waited for it to pass	281 (73.2)	249 (64.8)	
	Other	65 (16.9)	71 (18.5)	
Abdominal pain	Self-purchased OTC	307 (79.9)	207 (53.9)	<0.001
	Medications prescribed previously by a physician	84 (21.9)	70 (18.2)	
	Home methods	232 (60.4)	241 (62.8)	
	Immediately consulted the physician	81 (21.9)	175 (45.6)	
	Rested, stayed at home	76 (19.8)	128 (33.3)	
	Didn’t do anything special, just waited for it to pass	138 (35.9)	69 (18.0)	
	Other	25 (6.8)	12 (3.1)	
Urinary tract infection	Self-purchased OTC	323 (84.1)	230 (59.9)	<0.001
	Took an antibiotic available at home, recommended by a doctor for a previous infection/given by a friend	201 (52.3)	0 (0.0)	
	Medications prescribed previously by a physician	104 (27.1)	224 (58.3)	
	Home methods	137 (35.7)	53 (13.8)	
	Immediately consulted the physician	0 (0.0)	130 (33.9)	
	Rested, stayed at home	17 (4.4)	17 (4.4)	
	Didn’t do anything special, just waited for it to pass	45 (11.7)	54 (14.1)	
	Other	24 (6.2)	10 (2.6)	

### Time between self-medication and medical consultation

In the analysis of the average time that passes from the implementation of self-medication to the visit to the doctor, it was shown that in the case of abdominal pain, headache, urinary tract infection and rhinitis/cough/fever, both for children and adults, visits usually take place within 3 days. In the case of mental health disorders, adult patients most often reported themselves after more than a month, while caregivers with children earlier (*p* < 0.001). The exact summary and comparison of the visit time from the implementation of self-medication is presented in Table 5.

**TABLE 5:** Comparison of the time when patients report to the doctor from the self-medication implementation.

Self-medication cause	Czas	Adult N(%)	Child N(%)	*p*	V cramera
Headache	<3 days	190 (49.5)	308 (80.3)	<0.001	0.343
	3–7 days	105 (27.3)	52 (13.5)		
	8–14 days	55 (14.3)	14 (3.6)		
	14 days to month	11 (2.9)	9 (2.3)		
	> month	23 (6.0)	1 (0.3)		
Rhinitis, cough, sore throat	<3 days	260 (67.7)	353 (91.8)	<0.001	0.304
	3–7 days	114 (29.7)	29 (7.6)		
	8–14 days	9 (2.3)	1 (0.3)		
	14 days to month	1 (0.3)	1 (0.3)		
	> month	0 (0.0)	0 (0.0)		
Mental health disorders	<3 days	29 (7.6)	96 (25.0)	<0.001	0.292
	3–7 days	26 (6.7)	41 (10.7)		
	8–14 days	113 (29.4)	97 (25.3)		
	14 days to month	36 (9.4)	100 (26.0)		
	> month	180 (46.9)	50 (13.0)		
Abdominal pain	<3 days	236 (61.5)	325 (84.6)	<0.001	0.267
	3–7 days	104 (27.1)	38 (9.9)		
	8–14 days	24 (6.3)	14 (3.6)		
	14 days to month	12 (3.0)	6 (1.6)		
	> month	8 (2.1)	1 (0.3)		
Urinary tract infections	<3 days	246 (64.1)	346 (90.1)	<0.001	0.315
	3–7 days	121 (31.4)	33 (8.6)		
	8–14 days	15 (3.9)	3 (0.8)		
	14 days to month	1 (0.3)	2 (0.5)		
	> month	1 (0.3)	0 (0.0)		

N, number.

## Discussion

In our study, we hypothesized that physicians working in outpatient care, deal with self-medication or its negative effects in everyday practice, and are able to indicate the patterns of their patients’ behavior. Thus, we aimed to recognize and analyze the practical experience of outpatient physicians regarding self-medication of their adult and pediatric patients. We have indeed received abundant feedback showing both the causes and the frequencies for self-medication. The main three reasons for choosing self-medication in Poland according to surveyed doctors were: taking advice from other people - family members or friends (59.1%), finding information regarding treatment online (52.9%) and ability to self-medicate in this kind of symptoms/disease (51.6%). Failing to make an appointment with the physician was the fourth most common cause. When asked about the source of knowledge regarding used medication, the most often answers were: physician former recommendation (62.5%), member of family or friend recommendation (59.6%), own experience (49.0%) and finding suitable information online (46.9%). Data from the European Health Interview Survey (2006–2009) including 14 countries have shown that the mean self-medication prevalence in those years was 26.3%, being the highest in Poland (49.4%) (Brandão et al., 2020). The frequency of self-medication in developing countries varies from 60%–80% and is currently estimated for Europe at approximately 68% (Auta et al., 2012; Samuvel Babu et al., 2023). Interesting observations come from comparing our study with a report from India. Similarly to our observations, its authors found that one of the main sources of knowledge for practicing self-medication were the family members (Samuvel Babu et al., 2023).

Self-medication poses serious challenges for doctors. When a patient is commiting a misdiagnosis on the basis of similar symptoms in their family or loved ones, doctors have found it difficult to convince them of the error. Patients usually are convinced of the need for a specific treatment, often found on the Internet, which the doctor does not usually prescribe. In both of these situations, it is important to explain to the patient why the suspected disease or treatment is not appropriate, because despite the error, they will persist in their opinion and will not want to follow the treatment proposed by the doctor. Another danger of self-medication is sharing the medications with family members, which might be even life-threatening (Zanini et al., 2014). Excessive consumption of drugs, especially psychotropic drugs, is also a threat. As studies have shown, people with psychiatric illnesses tend to abuse prescribed drugs and develop addiction (Jeong et al., 2021; Martínez-León et al., 2023).

One of the forms of self-medication is the inappropriate use of antibiotics, which is associated with the administration of the wrong dose or treatment in the wrong time frame (Bi et al., 2023). Overuse of this group of drugs is associated with the emergence of pathogen resistance, especially in countries where there is a greater use of antibiotics. Research shows that about 57% of patients do not know that antibiotics do not affect viruses, therefore 44% are not aware that they are ineffective against cold or flu. Patients overestimate their knowledge of antibiotics, which can have a big impact on self-medication. In Poland, antibiotics are prescription drugs, but there are websites where they can be purchased without the prescription or where you can get a prescription without consulting a doctor. It is also common to use antibiotics prescribed by a doctor in the past, which the patient did not use or did not take the right dose (leftover antibiotics). Physicians also feel pressure from patients to prescribe antibiotics, despite the lack of indications for their use (Machowska and Stålsby Lundborg, 2018). In our study 72.1% of adults and nearly 40% of children caregivers used an antibiotic available at home recommended by a doctor for a previous infection because of the rhinitis, cough or sore throat, which are caused mainly by viral infections. The emerging problem of antibiotics misuse has been noticed especially among children under 3 years of age due to higher consumption of these drugs than in the general population (Alzahrani et al., 2018). A qualitative study from Spain found that antibiotics were misused both, by the children’s caregivers, as well as the physicians. Pediatricians declared that they felt to be forced to prescribe antibiotics as a rapid cure in unjustified circumstances. Parents’ self-medication was related to belief in the curative potential of antibiotics and the possibility of obtaining these drugs from pharmacies without prescription. At the same time physicians misused the antibiotics due to lack of education and the limited application of clinical guidelines (Arnau-Sánchez et al., 2023).

Numerous scientific studies show that increasing value is attached to the role of self-medication in supporting medical staff, which can improve clinical effects and relieve the burden on medical care systems (Dineen-Griffin et al., 2019). The role of the person educating the patient may be undertaken by a doctor, but also by other members of the medical staff (Ge et al., 2022). The introduction of pharmaceutical care in Poland opens the possibility of pharmaceutical consultations, and thus to solving minor health problems of the patient in the pharmacy. This would be possible thanks to the Minor Ailments Program, in which a pharmacist recognizes the disease, follows an appropriate treatment algorithm, and then offers the patient treatment with OTC or prescription drugs (Report, 2020).

## Summary

To sum up, the aim of this study was to recognize and analyze the practical experience of outpatient physicians regarding self-medication of their patients, as well as self-medication of children by their caregivers. This experience of outpatient physicians indicates that patients undertake self-treatment as a result of advice from family members or friends, relying on information regarding online treatment, and the patient’s own experience, which draws attention to the need for educational and organizational support for patients. According to doctors, the main sources of knowledge for patients when choosing drugs used in self-medication are physicians first, then family members or friends, their own experiences and information available online, which indicates the important role of a professional with medical education when deciding on the use of a drug. Further research is needed to assess whether the differences in antibiotics uptake may result from the awareness of the need to assess the indications for antibiotic therapy and the potential risks arising from the use of antibiotics, or from adults relying on their experience with the use of these drugs. The professional experience of doctors shows that patients are insufficiently involved in the area of ​​mental health. Most often, the lack of independent reaction on the part of patients and children’s caregivers to the deterioration of their mental condition was observed. Although in the case of children, almost 22% of doctors indicate that caregivers immediately consulted a doctor, for adults this percentage was only 5.7%. Side effects after self-medication are a common phenomenon observed in medical practice, and medical consultation is the most common form of seeking a solution to the problem by patients. Pharmacists were indicated as an important source of knowledge about the medicine used - according to doctors’ declarations, 43.2% of patients took advantage of such advice, which is an argument for increasing the role of pharmaceutical care within the Polish healthcare system.

The authors are aware of the limitations of this study, which undoubtedly include the methodology of collecting data using an online questionnaire. Nevertheless, CAWI-type tests are currently widely used in science. Firstly, they provide the opportunity to reach a wide group of recipients, increasing the scope of the research. Secondly, numerous studies have shown that people participating in anonymous Internet research have a greater predisposition to tell the truth and show lower levels of stress (Milton et al., 2017). Another limitation of the study is the lack of representativeness of the study group among all doctors in Poland, therefore it is necessary to conduct further research on a representative sample of doctors.

## Conclusions

Self-medication in Poland requires educational and organizational support at various levels - both social (information campaigns, school education), the healthcare system (increasing the role of medical professionals, including pharmacists), and finally in the area of ​​legislation. Two areas seem to be particularly alarming - the use of antibiotics by adults and children and the mental health of both populations. We recommend cooperation between scientific societies like Polish Society of Family Medicine and government entities to develop educational programs which will be an integral part of national and European health strategy. Allocation of financial resources for research on the effectiveness of various forms of self-treatment is strongly needed. As well as establishing cross-sectoral cooperation to promote self-medication as an element of public health.

## Data Availability

The raw data supporting the conclusions of this article will be made available by the authors, without undue reservation.
