# Supplementary material for: Self-medication of adults and children in Poland - results from outpatient health care physicians online questionnaire
Source: Front Pharmacol. 2024 Aug 13;15:1413811. doi: 10.3389/fphar.2024.1413811 (PMC11347341; doi:10.3389/fphar.2024.1413811)
Supplement: Supplementary file 1 [file DataSheet1.docx]

Dear Sirs,

we would like to invite you to participate in a survey aimed at assessing the phenomenon of self-medication by your patients.

Accordingly, we ask you to complete a fully anonymous survey. At any stage of the survey, you may opt out of further participation in the study without giving any reason. By completing the survey, you agree to take part in the study. The results obtained will be used to prepare a collective scientific study on the phenomenon of self-medication in Poland and a scientific publication.

The survey is conducted by the Polish Society of Family Medicine together with the Institute of Healthcare Management at Lazarski University.

Do you agree to participate in the study? **Yes/No**

1. **Sex**
   - Female
   - Male
   - I don't want to answer
2. **Year of bearth**
   - ________
3. **Indicate all the specializations you have**

○ I do not have a specialization

○ specialist in family medicine

○ specialist in internal medicine

○ specialist in pediatrics

○ a specialist in another field of medicine other than the above

○ in the course of specialization in family medicine

○ in the course of specialization in internal medicine

○ in the course of specialization in pediatrics

○ in the course of another specialization

1. **Indicate the size of the locality of the main place of work**
   - village
   - A city of up to 49,999 inhabitants
   - A city from 50 000 to 499 999 inhabitants
   - A city above 500 000 inhabitants
2. **Mark the main place of work**
   - GP
   - AOS
   - Other
3. **Indicate the province of the main place of work**
   - dolnośląskie
   - kujawsko-pomorskie
   - lubelskie
   - lubuskie
   - łódzkie
   - małopolskie
   - mazowieckie
   - opolskie
   - podkarpackie
   - podlaskie
   - pomorskie
   - śląskie
   - świętokrzyskie
   - warmińsko-Mazurskie
   - wielkopolskie
   - zachodniopomorskie
4. **Seniority**
   - ________
5. **What are the key problems adult patients turn to you with following an attempt to manage their ailments on their own**

○ headache

○ fever

○ sore and inflamed throat

○ muscle and joint pain

○ cough

○ runny nose

○ migraine

○ abdominal pain

○ back pain

○ digestive disorders (indigestion, diarrhea, constipation, etc.).

○ menstrual pain

○ inflammation of the urinary tract

○ allergy

○ lowered mood

○ other

1. **How long, on average, do adult patients use self-medication to relieve the following symptoms before they see a doctor**

[matrix with options to select 1 option for each symptom: less than 3 days, a week, 2 weeks, a month, more than a month].

○ headaches

○ cough, runny nose, sore throat

○ back/back pain

○ mental health problems

○ abdominal pain

○ urinary tract infection

1. **What methods did the patient use before attending the consultation**

**● to relieve headaches**

○ home remedies

○ used medicines purchased independently without a prescription

○ used medications prescribed by a doctor beforehand

○ rested, stayed at home

○ did nothing special, waited for it to pass on its own

○ other than those mentioned

○ the patient immediately sought medical attention

○ to relieve the respiratory tract infection.

○ home remedies

○ used medicines purchased independently without a prescription

○ used medicines prescribed by a doctor beforehand

○ took an antibiotic, available at home, prescribed by a doctor for a previous infection / given by a friend

○ rested, stayed at home

○ did nothing special, waited for it to pass on its own

○ other than those mentioned

○ the patient immediately sought medical attention

**● to relieve back/back pain.**

○ home remedies

○ used medicines purchased independently without a prescription

○ used medications prescribed by a doctor beforehand

○ rested, stayed at home

○ did nothing special, waited for it to pass on its own

○ other than those mentioned

○ the patient immediately went to the doctor.

**● to alleviate mental health problems**

○ home remedies

○ used medicines purchased independently without a prescription

○ used medications prescribed by a doctor beforehand

○ rested, stayed at home

○ did nothing special, waited for it to pass on its own

○ other than those mentioned

○ the patient immediately sought medical attention

**● to relieve abdominal pain**

○ home remedies

○ used medicines purchased independently without a prescription

○ used medications prescribed by a doctor beforehand

○ rested, stayed at home

○ did nothing special, waited for it to pass on its own

○ other than those mentioned

○ the patient immediately sought medical attention

○ to alleviate the urinary tract infection.

○ home remedies (e.g., infusions, garlic, honey, compresses, exercise, etc.).

○ used medicines bought on their own without a prescription

○ used medications prescribed by a doctor beforehand

○ took an antibiotic, available at home, prescribed by a doctor for a previous infection / given by a friend

○ rested, stayed at home

○ did nothing special, waited for it to pass on its own

○ other than those mentioned

○ the patient immediately reported to the doctor

1. **What key health problems in their children do their parents/guardians approach you with**

○ headache

○ fever

○ sore and inflamed throat

○ muscle and joint pain

○ cough

○ runny nose

○ abdominal pain

○ digestive system disorders (indigestion, diarrhea, constipation, etc.).

○ menstrual pain

○ inflammation of the urinary tract

○ allergy

○ lowered mood

○ other

1. **How long, on average, do parents/guardians use methods to relieve the following symptoms in their child on their own, before reporting them to the doctor?**

[matrix with options to select 1 option for each symptom: less than 3 days, a week, 2 weeks, a month, more than a month].

○ headaches

○ cough, runny nose, sore throat

○ back/back pain

○ mental health problems

○ abdominal pain

○ allergies

○ urinary tract infections

1. **What methods do parents/guardians typically use before seeking consultation**

**○ to relieve their child's headaches**

■ home remedies

■ used medicines bought on their own without a prescription

■ used medications prescribed by a doctor beforehand

■ child/children rested, stayed at home

■ did nothing special, waited for it to pass on its own

■ other than those mentioned

■ most parents/guardians immediately go to the doctor with their child.

**○ in order to relieve their child's symptoms of respiratory tract infection.**

■ home remedies

■ used medicines bought on their own without a prescription

■ used medications prescribed by a doctor beforehand

■ administered an antibiotic, available at home, recommended by a doctor for a previous infection / given by a friend

■ child/children rested, stayed at home

■ do nothing special, wait for it to pass on its own

■ other than those mentioned

■ most parents/guardians go to the doctor with their child right away

**○ in order to relieve their child's back/back pain.**

■ home remedies (e.g., infusions, garlic, honey, compresses, exercise, etc.).

■ used medicines bought on their own without a prescription

■ used medicines prescribed by a doctor beforehand

■ the child/children have rested, stayed at home

■ did nothing special, waited for it to pass on its own

■ other than those mentioned

■ most parents/guardians immediately go to the doctor with their child.

**○ In order to alleviate their child's mental health problems**

■ home remedies

■ used medications purchased on their own without a prescription

■ used medication prescribed by a doctor beforehand

■ child/children rested, stayed at home

■ have not done anything special, waiting for it to pass on its own

■ other than those mentioned

■ most parents/guardians immediately go to the doctor with their child.

**○ in order to alleviate their child's digestive disorder (abdominal pain).**

■ home remedies (e.g., infusions, garlic, honey, compresses, exercise, etc.).

■ used medicines bought on their own without a prescription

■ used medicines prescribed by a doctor beforehand

■ the child/children have rested, stayed at home

■ did nothing special, waited for it to pass on its own

■ other than those mentioned

■ most parents/guardians immediately go to the doctor with their child.

**○ in order to alleviate their child's allergy symptoms.**

■ home remedies (e.g., infusions, garlic, honey, compresses, exercise, etc.).

■ used medicines bought on their own without a prescription

■ used medications prescribed by a doctor beforehand

■ the child/children have rested, stayed at home

■ did nothing special, waited for it to pass on its own

■ other than those mentioned

■ Most parents/guardians immediately go to the doctor with their child.

1. **Why do patients/parents/guardians choose to self-medicate and do not choose to seek medical advice?**

○ they failed to make an appointment, the doctor was unavailable, they had to wait too long

○ usually in this type of illness/illness they can treat the child/children themselves

○ the symptoms stopped before they thought a consultation would be useful

○ in this case, the advice of a pharmacist was enough for them.

○ other people: family, friends, advised them on treatment

○ they found information about treatment options on the Internet

○ other cause

1. **What usually prompts patients/parents/guardians to seek consultation with you**

○ symptoms were more severe than usual

○ self-treatment was unsuccessful

○ there just happened to be a free appointment and I took advantage of it

○ I did not know how to self-treat.

○ other people: family, friends, advised me to go to a doctor

○ other reason

1. **How do patients/parents/guardians know what medications to use when faced with an emerging health problem?**

○ they always help them in such a situation

○ they were once recommended to them by a doctor and since then I have been buying them myself

○ they were recommended them by a pharmacist

○ they were recommended them by a family member/friend

○ they were convinced by an advertisement

○ they found information about the treatment on the Internet

○ other

1. **What kind of sources of knowledge about treatment on the Internet do patients/parents/guardians use most often?**

○ drug manufacturer websites

○ blogs dedicated to treatment

○ specialized sites devoted to medical advice

○ online forums

○ articles on news portals

○ other, not mentioned above

1. **Have you encountered in your practice in the last two years any adverse reactions to self-medication?**

○ yes

○ no

○ don't remember

1. **What do patients/parents/guardians most often do when an adverse reaction occurs?**

○ they report to the doctor

○ they report to the pharmacist

○ they report to the drug manufacturer

○ they report to the Office for Registration of Medicinal Products, Medical Devices and Biocidal Products

○ they discontinue the drug

○ they act differently than indicated above

○ do nothing, wait until the side effect resolves on its own
